# A Strong Correlation Between Pleural Fluid and Serum C-Reactive Protein Levels Across a Spectrum of Pleural Effusions

**DOI:** 10.14740/jocmr6513

**Published:** 2026-04-15

**Authors:** Majed Odeh, Yana Kogan, Edmond Sabo

**Affiliations:** a Department of Internal Medicine A, Bnai Zion Medical Center, Haifa, Israel; b Faculty of Medicine, Technion–Israel Institute of Technology, Haifa, Israel; c Pulmonary Division, Carmel Medical Center, Haifa, Israel; d Institute of Pathology, Carmel Medical Center, Haifa, Israel; e These authors contributed equally to the work.

**Keywords:** C-reactive protein, Pleural effusion, Transudative effusion, Parapneumonic effusion, Malignant effusion, Tuberculous effusion, Correlation

## Abstract

**Background:**

C-reactive protein (CRP) is a key acute-phase reactant, primarily synthesized by hepatocytes and released into the bloodstream. Both serum CRP (CRPs) and pleural fluid CRP (CRPpf) have been shown to aid in distinguishing between different types of pleural effusion (PE). As CRPpf is largely derived from CRPs, a strong correlation between their levels is expected. However, limited data exist regarding this relationship, and no previous studies have compared the strength of this correlation across different PE etiologies. This retrospective study aimed to evaluate the correlation between CRPpf and CRPs levels in various PE types and, for the first time, to compare the strength of this association between groups.

**Methods:**

A total of 492 patients with PE were included: 210 with transudative PE (TrPE), 86 with uncomplicated parapneumonic effusion (UCPPE), 60 with complicated parapneumonic effusion (CPPE), 126 with malignant PE (MPE), and 10 with tuberculous PE (TPE). Data are presented as mean ± standard deviation.

**Results:**

Mean CRPs and CRPpf levels, respectively, were as follows: TrPE (11.3 ± 5.7 mg/L; 4.6 ± 2.8 mg/L), UCPPE (145.3 ± 67.6 mg/L; 58.5 ± 38.5 mg/L), CPPE (302.2 ± 75.6 mg/L; 112 ± 65 mg/L), MPE (56.1 ± 39.5 mg/L; 18.9 ± 13.9 mg/L), and TPE (98.7 ± 12.9 mg/L; 45.0 ± 9.4 mg/L). A statistically significant positive correlation between CRPpf and CRPs was observed in all groups: TrPE (r = 0.81, P < 0.0001), UCPPE (r = 0.90, P < 0.0001), CPPE (r = 0.57, P < 0.0001), MPE (r = 0.81, P < 0.0001), and TPE (r = 0.91, P < 0.0001). The correlation was significantly stronger in the UCPPE and TPE groups compared to the others, while the CPPE group showed the weakest correlation. Correlation strength in the TrPE and MPE groups was intermediate, but significantly greater than that in CPPE.

**Conclusions:**

A strong and statistically significant correlation between CRPpf and CRPs levels exists across all major types of PE. The varying strength of this correlation among groups—highest in UCPPE and TPE, and lowest in CPPE—may reflect the influence of local pleural factors, such as inflammation, cellular injury, local CRP synthesis, and lymphatic drainage impairment, on pleural CRP levels.

## Introduction

C-reactive protein (CRP), a member of the pentraxin family, is recognized as one of the most clinically significant acute-phase reactants. It is primarily synthesized by hepatocytes in the liver. In response to inflammatory stimuli—most commonly infections, autoimmune disorders, malignancies, and tissue injury such as burns, trauma, or fractures—CRP production and release into the circulation increase markedly within hours [1, 2]. Serum CRP (CRPs) levels can peak within approximately 48 h and may rise dramatically, sometimes exceeding a 1,000-fold increase, depending on the intensity of the inflammatory or tissue-damaging process. This acute-phase response is predominantly mediated by pro-inflammatory cytokines, especially interleukin-6 (IL-6), as well as tumor necrosis factor-α (TNF-α), interleukin-1β (IL-1β), and interleukin-17 (IL-17) [1, 2].

The diagnostic value of CRPs and pleural fluid CRP (CRPpf) in differentiating among the various causes of pleural effusion (PE) remains an active area of investigation [3–15]. Several studies have demonstrated that both CRPs and CRPpf can aid in distinguishing transudative PE (TrPE) from exudative PE (ExPE), as well as in differentiating between specific subtypes of ExPE [3–60]. Given that circulating blood is the primary source of CRP in pleural fluid [1, 2, 14, 18, 21, 44], a strong correlation between CRPpf and CRPs would be expected. However, available data on the strength and consistency of this correlation are limited [21, 26, 38], and to date, no study has systematically compared this relationship across different etiologies of PE.

This retrospective study aimed to evaluate the correlation between CRPpf and CRPs levels in patients with PE of varying etiologies. To the best of our knowledge, this is the first study to compare the strength of this correlation across different diagnostic groups.

## Materials and Methods

The study population comprised 492 patients diagnosed with PE. Among them, 210 patients (aged 33–96 years) had TrPE, primarily due to congestive heart failure (CHF) (n = 200), with fewer cases attributed to liver cirrhosis (n = 8) and nephrotic syndrome (n = 2). Additionally, 86 patients (aged 24–91 years) presented with uncomplicated parapneumonic effusion (UCPPE), 60 (aged 30–91 years) with complicated PPE (CPPE), 126 (aged 29–95 years) with malignant pleural effusion (MPE), and 10 (aged 23–86 years) with tuberculous pleural effusion (TPE). Patients with comorbidities or medications that may significantly influence levels CRPs or CRPpf, and the results of our study, were not included in the study.

TrPE was attributed to CHF when all of the following criteria were met: 1) cardiomegaly; 2) clinical, echocardiographic evidence of cardiac dysfunction; 3) radiographic signs of pulmonary congestion and/or peripheral edema; 4) resolution of the effusion with appropriate CHF treatment; and 5) absence of alternative etiologies. TrPE was considered secondary to liver cirrhosis or nephrotic syndrome only when a definitive diagnosis of the underlying condition was established and no other cause of PE was identified. UCPPE was diagnosed in patients presenting with an acute febrile illness, purulent sputum, radiographic evidence of pulmonary infiltrates, and a favorable response to antibiotic therapy, without evidence of bacterial invasion into the pleural space or other identifiable causes of PE. CPPE was diagnosed in the presence of clinically and radiographically confirmed pneumonia, with at least one of the following indicators of pleural space infection: positive Gram stain or culture of pleural fluid, presence of loculations, pleural thickening, pleural fluid pH < 7.2, empyema, or an effusion occupying ≥ 50% of the hemithorax. MPE was defined by the presence of malignant cells on cytological examination of pleural fluid or histological analysis of pleural biopsy specimens, in the absence of other potential causes of PE. TPE was diagnosed based on either a positive culture for *Mycobacterium tuberculosis* (from pleural fluid, sputum, or biopsy specimens), a positive Ziehl-Neelsen stain for acid-fast bacilli, or histological evidence of caseating granulomas on pleural biopsy, with no other explanatory cause for the effusion and the caseating granulomas.

Relevant data were extracted from patients’ medical records. Only those with a definitive diagnosis of PE (classified as TrPE, UCPPE, CPPE, MPE, or TPE) and available measurements of CRPs and CRPpf levels were included.

CRPs and CRPpf concentrations were determined using a Cobas c 501 analyzer (Roche Diagnostics), following the manufacturer’s guidelines. The study protocol was approved by the Institutional Ethics Committee of Bnai Zion Medical Center (Protocol No.: 0107-16-BNZ) and conducted in accordance with the Declaration of Helsinki.

Statistical analyses were conducted using IBM SPSS Statistics, version 23 (Armonk, NY, USA). Continuous data are reported as mean ± standard deviation (SD), accompanied by 95% confidence intervals (CIs). The distribution of variables was evaluated using the Kolmogorov–Smirnov test to assess normality. Correlations between CRPpf and CRPs levels within each group were evaluated using Pearson’s correlation coefficient. To compare the strength of these correlations across groups, the Fisher r-to-z transformation was applied. A P value of ≤ 0.05 was considered indicative of statistical significance.

## Results

The TrPE group comprised 210 patients with a mean age of 77.3 ± 10.4 years. The UCPPE group included 86 patients (65.9 ± 18.1 years), the CPPE group 60 patients (74.1 ± 13.6 years), the MPE group 126 patients (75.9 ± 10.2 years), and the TPE group 10 patients (66.1 ± 19.2 years) (Table 1). Across all groups, CRPs levels consistently exceeded CRPpf levels. The TrPE group was significantly older than both the UCPPE and TPE groups (P < 0.0001), but showed no significant age difference compared to the CPPE and MPE groups (P > 0.05). Patients in the MPE group were significantly older than those in the UCPPE and TPE groups (P < 0.001), as were patients in the CPPE group (P < 0.003) (Table 1).

**Table 1:** Age and CRP Levels (CRPs and CRPpf) Across TrPE, UCPPE, CPPE, MPE, and TPE Groups

Variable	TrPE (n = 210)	UCPPE (n = 86)	CPPE (n = 60)	MPE (n = 126)	TPE (n = 10)
Age (years), mean ± SD	77.3 ± 10.4^a^	65.9 ± 18.1	74.1 ± 13.6^b^	75.9 ± 10.2^c^	66.1 ± 19.2
CRPs (mg/L), mean ± SD	11.3 ± 5.7	145.3 ± 67.6	302.2 ± 75.6	56.1 ± 39.5	98.7 ± 12.9
95% CI	10.5–12.1	130.5–160.1	283.9–320.4	23.3–71.0	91.4–107.9
CRPpf (mg/L), mean ± SD	4.6 ± 2.8	58.5 ± 38.5	112.0 ± 65.0	18.9 ± 13.9	45.0 ± 9.4
95% CI	4.2–5.0	19.2–85.1	96.0–128.0	7.6–28.1	8.3–51.7

^a^P < 0.0001 versus UCPPE and TPE;^ b^P < 0.003 versus UCPPE and TPE; ^c^P < 0.001 versus UCPPE and TPE. SD: standard deviation; CRP: C-reactive protein; CI: confidence interval; CRPs: serum CRP; CRPpf: pleural fluid CRP; TrPE: transudative pleural effusion; UCPPE: uncomplicated parapneumonic effusion; CPPE: complicated parapneumonic effusion; MPE: malignant pleural effusion; TPE: tuberculous pleural effusion.

**Figure 1:**
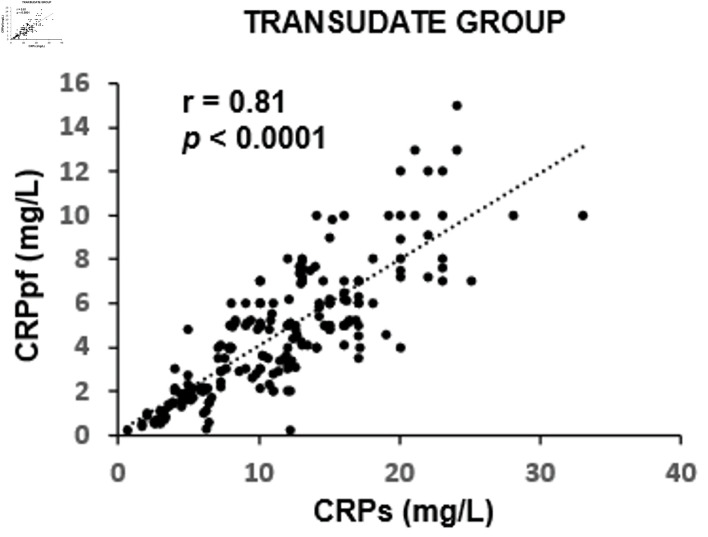
Correlation between CRPpf and CRPs in the TrPE group. CRPs: serum C-reactive protein; CRPpf: pleural fluid C-reactive protein; r: correlation coefficient; TrPE: transudative pleural effusion.

**Figure 2:**
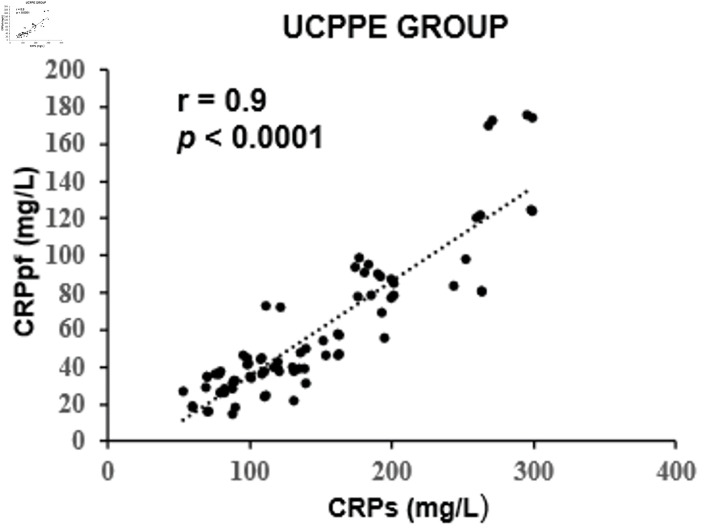
Correlation between CRPpf and CRPs in the UCPPE group. UCPPE: uncomplicated parapneumonic effusion; CRPs: serum C-reactive protein; CRPpf: pleural fluid C-reactive protein; r: correlation coefficient.

**Figure 3:**
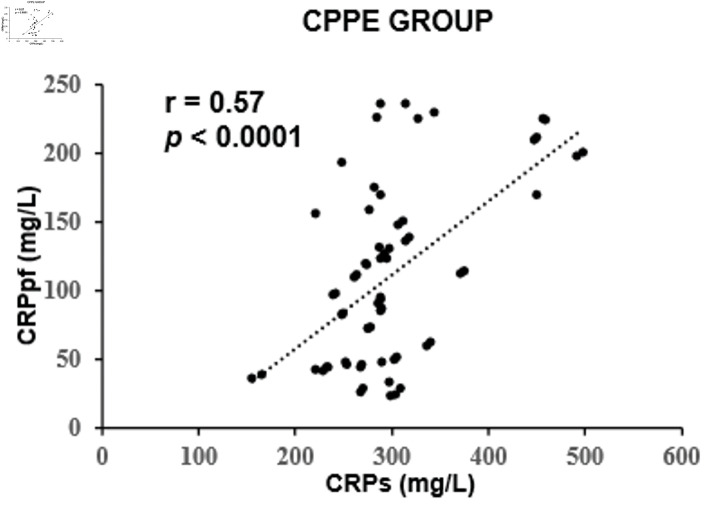
Correlation between CRPpf and CRPs in the CPPE group. CPPE: complicated parapneumonic effusion; CRPs: serum C-reactive protein; CRPpf: pleural fluid C-reactive protein; r: correlation coefficient.

**Figure 4:**
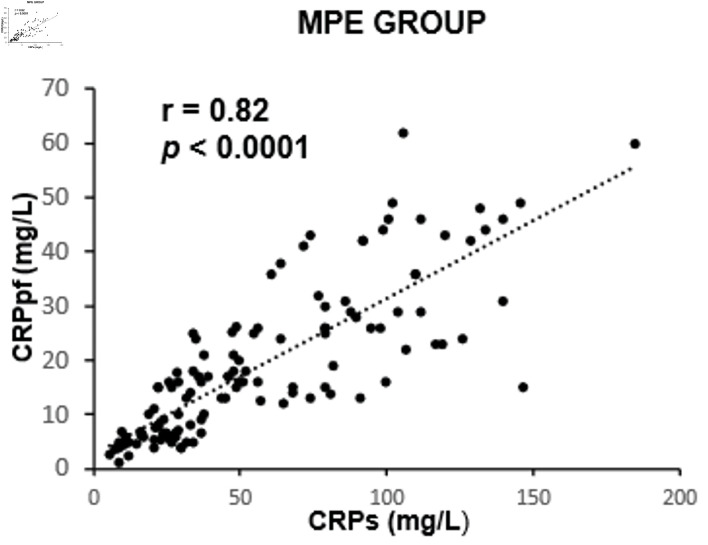
Correlation between CRPpf and CRPs in the MPE group. MPE: malignant pleural effusion; CRPs: serum C-reactive protein; CRPpf: pleural fluid C-reactive protein; r: correlation coefficient.

**Figure 5:**
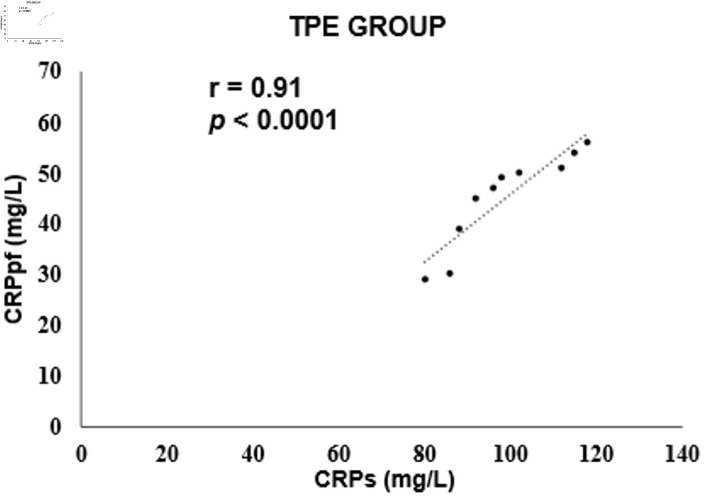
Correlation between CRPpf and CRPs in the TPE group. TPE: tuberculous pleural effusion; CRPs: serum C-reactive protein; CRPpf: pleural fluid C-reactive protein; r: correlation coefficient.

The mean concentrations of CRPs and CRPpf were 11.3 ± 5.7 mg/L and 4.6 ± 2.8 mg/L, respectively, in the TrPE group; 145.3 ± 67.6 mg/L and 58.5 ± 38.5 mg/L in the UCPPE group; 302.2 ± 75.6 mg/L and 112 ± 65 mg/L in the CPPE group; 56.1 ± 39.5 mg/L and 18.9 ± 13.9 mg/L in the MPE group; and 98.7 ± 12.9 mg/L and 45.0 ± 9.4 mg/L in the TPE group (Table 1). In each group, CRPpf levels were significantly and positively correlated with corresponding CRPs values: TrPE (r = 0.81, P < 0.0001) (Fig. 1), UCPPE (r = 0.90, P < 0.0001) (Fig. 2), CPPE (r = 0.57, P < 0.0001) (Fig. 3), MPE (r = 0.81, P < 0.0001) (Fig. 4), and TPE (r = 0.91, P < 0.0001) (Fig. 5).

Intergroup comparisons revealed that the correlation coefficient in the TrPE group was significantly higher than in the CPPE group (P < 0.001), but not significantly different from the MPE group (P = 0.79). The UCPPE group demonstrated a significantly stronger correlation than the TrPE (P < 0.01), CPPE (P < 0.0001), and MPE groups (P = 0.029), with no significant difference compared to the TPE group (P = 0.78). Additionally, the MPE group showed a significantly higher correlation coefficient than the CPPE group (P < 0.001). The TPE group exhibited significantly stronger correlations than the TrPE (P < 0.01), CPPE (P < 0.0001), and MPE groups (P < 0.01) (Table 2).

**Table 2:** Correlation Between Levels of CRPpf and CRPs for Various Groups of PE With Its Correlation Coefficient (r) and Its Statistical Significance (P)

Variable	TrPE (n = 210)	UCPPE (n = 86)	CPPE (n = 60)	MPE (n = 126)	TPE (n = 10)
r	0.81^a^	0.9^b, c, d^	0.57	0.82^a^	0.91^c, e^
P	< 0.0001	< 0.0001	< 0.0001	< 0.0001	< 0.0001

^a^P < 0.001 versus CPPE, ^b^P = 0.009 versus TrPE, ^c^P < 0.0001 versus CPPE, ^d^P = 0.029 versus MPE, ^e^P < 0.01 versus TrPE and MPE. CRPs: serum CRP; CRPpf: pleural fluid CRP; TrPE: transudative pleural effusion; UCPPE: uncomplicated parapneumonic effusion; CPPE: complicated parapneumonic effusion; MPE: malignant pleural effusion; TPE: tuberculous pleural effusion.

## Discussion

The findings of the present study revealed a significant positive correlation between CRPpf and CRPs across all five diagnostic groups (Table 1, Figs. 1–5). The strongest correlations were observed in the TPE and UCPPE groups (r = 0.91, P < 0.0001 and r = 0.90, P < 0.0001, respectively), followed by the MPE and TrPE groups (r = 0.82, P < 0.0001 and r = 0.81, P < 0.0001, respectively). The weakest correlation was found in the CPPE group (r = 0.57, P < 0.0001).

To date, only three studies have specifically examined the correlation between CRPpf and CRPs [21, 26, 38]. One study analyzed a combined cohort of 72 patients with TrPE and ExPE, reporting a significant correlation (r = 0.69, P < 0.0001) [21]. Another study assessed the relationship separately in patients with TrPE (n = 12) and ExPE (n = 36), finding a statistically significant correlation only in the ExPE group (r = 0.49, P = 0.002), while the TrPE group showed a weaker, non-significant association (r = 0.3, P = 0.35) [38]. A third investigation reported correlation coefficients of r = 0.64 in TrPE (n = 67), r = 0.54 in PPE (including both UCPPE and CPPE; n = 48), r = 0.80 in MPE (n = 72), and r = 0.74 in TPE (n = 67). However, P values were not provided for these correlations [26]. In comparison to these previous reports, our results diverge, except for the findings related to the MPE group. Given the larger size of our study population—approximately double that of the aforementioned studies—our results may offer a more robust and reliable estimation of the correlation between CRPpf and CRPs across different types of PE.

To our knowledge, this study is the first to compare the strength of correlation coefficients between CRPpf and CRPs levels across different types of PE. The analysis demonstrated that the UCPPE and TPE groups exhibited similarly strong correlations, which were significantly higher than those observed in the other groups (Table 2). Likewise, the correlation strengths in the TrPE and MPE groups were comparable to each other and significantly greater than that observed in the CPPE group (Table 2). As this comparative analysis has not been previously reported, there are no available data in the current medical literature to account for the observed differences in correlation strength among the various PE groups. Nevertheless, several potential explanations may be proposed to account for these findings.

Given that CRPpf primarily originates from CRPs, which is predominantly synthesized and secreted by the liver [1, 2], significant differences in the correlation strength between CRPpf and CRPs across various PE types would not be expected—unless additional, non-hepatic sources of CRP contribute to its pleural fluid concentration. Indeed, although the liver is the principal source of circulating CRP, limited local production has been documented in various cell types, including macrophages, lymphocytes, lung epithelial cells, vascular smooth muscle cells, endothelial cells, adipocytes, and certain neuronal cells [1, 2, 61–69]. The regulatory mechanisms underlying CRP expression by these cells remain incompletely understood. Importantly, such local production does not significantly impact CRPs levels [1, 2, 61–69], but may contribute to CRPpf concentrations, particularly in conditions characterized by intense local inflammation.

CPPE is associated with markedly increased pleural capillary permeability due to elevated levels of proinflammatory cytokines such as TNF-α and IL-8. This results in the exudation of protein-rich plasma and inflammatory cells—predominantly neutrophils—into the pleural space [70–73]. CPPE is distinguished by a higher degree of inflammation and tissue injury within the pleura, pleural cavity, and adjacent pulmonary tissue compared to other PE types. It also features elevated absolute numbers of lymphocytes and macrophages at the site of inflammation [60, 70–73]. Although neutrophils do not produce CRP, macrophages and lymphocytes, along with activated lung epithelial, endothelial, and vascular smooth muscle cells, may locally synthesize CRP in response to inflammatory stimuli [1, 2, 61–69], potentially increasing CRPpf levels in CPPE. Conversely, the extensive tissue damage and cellular lysis characteristic of CPPE may lead to the release of intracellular contents into the pleural space. Since CRP is not typically an intracellular protein [1, 2], this lysis may dilute CRP concentrations in the pleural fluid. Furthermore, impaired pleural lymphatic drainage—commonly seen in CPPE due to inflammatory damage [70–76]—can lead to pleural fluid accumulation and dilution of soluble mediators, including CRP. Collectively, while local CRP production in CPPE might increase CRPpf levels, this effect may be offset by dilutional factors resulting from increased fluid volume and cellular breakdown. The net effect appears to be a relative decrease in CRPpf levels, which could explain the weaker correlation observed between CRPpf and CRPs in CPPE compared to other PE subtypes.

UCPPE, like CPPE, results from increased pleural capillary permeability triggered by proinflammatory cytokines, leading to the exudation of protein- and neutrophil-rich plasma into the pleural space [70–73, 76–79]. However, UCPPE is typically associated with sterile pleural fluid and a markedly lower degree of inflammation and tissue injury affecting the pleura, pleural cavity, and adjacent lung parenchyma compared to CPPE [60, 70–73, 76–79]. Consequently, the extent of cellular lysis and the resulting release of intracellular contents into the pleural space, as well as the damage and obstruction of pleural lymphatic drainage pathways, are considerably less pronounced [70–74]. Although both cell lysis and impaired lymphatic absorption can contribute to a reduction in CRPpf levels, in UCPPE, these effects are likely modest. Moreover, any potential decrease in CRPpf due to such local factors may be counterbalanced by limited local CRP synthesis by inflammatory and structural cells present in the affected tissues. Overall, the relatively mild local pathology in UCPPE appears to minimize confounding influences on CRPpf concentration. As a result, CRPpf levels in this group are more directly reflective of CRPs concentrations, which may explain the strong correlation observed between CRPpf and CRPs in UCPPE.

From a pathophysiological perspective, TPE is perhaps the most similar to UCPPE. Like UCPPE, TPE commonly presents as a sterile exudate and is driven by increased pleural capillary permeability secondary to proinflammatory cytokine activity, resulting in protein-rich plasma leakage into the pleural space. In its early (hyperacute) phase, TPE may also exhibit neutrophil predominance, similar to UCPPE, which later transitions to a lymphocyte-dominant profile. Overall, the intensity of inflammation in TPE is comparable to that observed in UCPPE [80–85]. However, several distinguishing features set TPE apart. Unlike UCPPE, TPE involves the formation of caseating granulomas within the pleura and is associated with diffuse pleural inflammation, triggered primarily by mycobacterial antigens, and less frequently by the bacilli themselves [80–85]. This granulomatous response may further increase macrophage infiltration into the pleural space. The combined presence of activated macrophages and lymphocytes, both stimulated by inflammatory cytokines, could contribute to local CRP production at a potentially higher level than that seen in UCPPE [80–85]. This, in turn, may elevate CRP concentrations in the pleural fluid. Conversely, the extensive granulomatous inflammation may also result in enhanced cellular lysis, releasing additional fluid into the pleural cavity and contributing to a dilutional effect on CRPpf levels [80–85]. Furthermore, the diffuse lymphocytic pleuritis characteristic of TPE may cause greater damage and obstruction to the pleural lymphatics—key structures involved in pleural fluid resorption—than is typically observed in UCPPE [70–73, 76, 80–85]. The resulting accumulation of pleural fluid could further reduce CRPpf concentration due to dilution. Taken together, while local cellular production of CRP in TPE may increase CRPpf levels, this effect appears to be counterbalanced by the dilutional influences of increased pleural fluid volume. The net result is that CRPpf in TPE, similar to UCPPE, largely reflects systemic CRP levels rather than being significantly altered by local pleural factors. This is consistent with the strong correlation between CRPpf and CRPs observed in the TPE group in our study.

In MPE, the degree of inflammation is generally lower than that observed in CPPE, UCPPE, and TPE [73–76, 86–89], a finding supported in our study by the comparatively lower levels of both CRPs and CRPpf in the MPE group. However, tissue injury and cellular lysis within the pleura and pleural space—driven by malignant cell infiltration—appear to be more pronounced in MPE than in UCPPE and TPE, though less severe than in CPPE [73–76, 86–89]. Additionally, the high turnover rate of malignant cells, characterized by rapid proliferation and death, contributes further to the overall burden of cellular lysis in MPE. This extensive cell breakdown in the pleura, pleural cavity, and surrounding lung tissue releases intracellular contents into the pleural space, potentially diluting CRP concentrations. The effect is compounded by impaired pleural fluid absorption, commonly due to obstruction of lymphatic channels by malignant cells [73–76, 86–89]. While local CRP production by inflammatory and structural cells—such as lymphocytes, macrophages, lung epithelial cells, and vascular smooth muscle and endothelial cells—may modestly elevate CRPpf, the dilutional effects from cellular lysis and reduced lymphatic clearance likely outweigh this contribution. As a result, CRPpf levels in MPE are influenced by local pathophysiological processes, leading to a diminished correlation with CRPs. Nonetheless, this reduction in correlation strength is less pronounced than that observed in CPPE, where tissue destruction, cellular breakdown, and lymphatic obstruction are considerably more extensive [73–76, 86–89].

Unlike the other four groups, patients with TrPE do not exhibit an inflammatory process, tissue damage, or significant cellular lysis in the pleura, pleural cavity, or adjacent lung tissue [73–76, 86]. Therefore, in these patients, the concentration of CRPpf is expected to reflect solely CRPs level, and the correlation between CRPpf and CRPs should be the strongest. Our findings did demonstrate a strong correlation in the TrPE group—significantly stronger than that observed in CPPE. However, this correlation was similar to that found in MPE and significantly weaker than in both UCPPE and TPE. To interpret this result, it is important to consider that our TrPE patients were undergoing treatment with diuretics of varying dosages and durations. It is well known that, according to Light’s criteria, approximately 25% of patients receiving high-dose or long-term diuretic therapy may have their TrPE misclassified as an exudate [86, 90–94]. This misclassification is primarily due to an increase in the pleural fluid/serum protein ratio to > 0.5, which results from diuretics removing more solvent (water) than solute (protein) from the PE [86, 90–94]. This mechanism likely led to an elevated CRPpf concentration in a subset of our TrPE patients, thereby reducing the strength of the correlation between CRPpf and CRPs in this group. This reduction in correlation strength was similar to that seen in MPE but remained significantly greater than in CPPE. Thus, the TrPE group, in terms of correlation strength, occupied an intermediate position—comparable to MPE and between the stronger correlations observed in UCPPE and TPE and the weaker correlation seen in CPPE.

The mean age of the TrPE, CPPE, and MPE groups was significantly higher than that of the UCPPE and TPE groups (Table 1). Although CRPs levels may increase with age by approximately 1 mg/L [95–98], this increase is minimal and typically remains within the normal range (< 5 mg/L). Such a small increment is unlikely to significantly affect comparisons of CRPs or CRPpf levels among different groups of PE, including those examined in our study, in which the mean CRPs and CRPpf levels were substantially higher than 1 mg/L. This is even more evident when considering the comparison of correlations between CRPpf and CRPs across different PE groups (the aim of our present study). Because CRPpf is primarily derived from CRPs, any increase in CRPs would be accompanied by a corresponding increase in CRPpf. Therefore, the correlation between CRPpf and CRPs levels is unlikely to be meaningfully influenced by small age-related increases in CRPs. This argument also applies to other potential covariates, such as gender [99–102], body mass index [103–106], and smoking [107–110].

This study has two main limitations. First, its retrospective design may introduce inherent biases related to data collection and analysis. Second, the sample size TPE group is too small for robust statistical inference. This main limitation was unavoidable because tuberculosis and tuberculous pleurisy are uncommon in our country. However, statistically, the results are meaningful since the correct statistical test was applied, providing a significant P value. At least, although weak, this statistical significance stimulates performing similar studies on large cohorts of subjects in regions where tuberculous pleurisy is common, in order to validate this statistical significance and its clinical meaningfulness.

### Conclusions

This study demonstrated a significant positive correlation between CRPpf and CRPs levels across all five PE groups, a finding consistent with the understanding that CRPs is the primary source of CRPpf. Compared to the limited existing studies on this topic, our results appear more robust and comprehensive. Notably, this is the first study to compare the strength of this correlation across different PE etiologies. We found the strongest associations in the UCPPE and TPE groups, and the weakest in the CPPE group. These variations suggest that local pleural factors—such as cellular CRP production, tissue lysis, and impaired fluid resorption—may influence CRPpf levels independently of CRPs. While plausible mechanistic explanations for these differences have been discussed in detail above, they remain, to some extent, hypothetical. These findings highlight the need for further research—both prospective and retrospective—in larger and more diverse patient populations to confirm and expand upon our observations.

## Data Availability

Data supporting the findings of this study are available from the corresponding author upon reasonable request.

## Abbreviations

CRP: C-reactive protein
CRPs: serum C-reactive protein
CRPpf: pleural fluid C-reactive protein
PE: pleural effusion
TrPE: transudative pleural effusion
UCPPE: uncomplicated parapneumonic effusion
CPPE: complicated parapneumonic effusion
MPE: malignant pleural effusion
TPE: tuberculous pleural effusion
IL-6: interleukin-6
TNF-α: tumor necrosis factor-α
IL-1β: interleukin-1β
IL-17: interleukin-17
ExPE: exudative pleural effusion
CHF: congestive heart failure
SD: standard deviation
CI: confidence interval
IL-8: interleukin-8
